# On Ovarian Irritation

**Published:** 1852-01

**Authors:** Fleetwood Churchill


ARTICLE IV.

*On Ovarian Irritation.* By FLEETWOOD CHURCHILL, M. D.

The following description relates to an affection which, although very common, is but little noticed in books. This is probably arisen from its having been placed among the symptoms of other diseases, although it is quite distinguishable from them.

It resembles most closely the disease described by Dr. Tilt, under the name of subacute ovaritis ; but the cases I have seen led me to differ from that very intelligent writer, and to conclude that the affection to which I refer is not inflammatory. I have, therefore, preferred the term *Ovarian Irritation.*

I have met with it in women of all ages between the commencement and cessation of menstruation, so that I do not think age has much influence in the production of the disease ; but I am quite certain that it is most frequent in women of a delicate, nervous temperament, though by no means confined to them.

The chief characteristic symptom is an uneasiness, amounting in the greater number of cases to pain, and in some cases to very severe pain, in one or both iliac or inguinal regions, but most frequently in the left, which Prof. Simpson seems to think is owing to the propinquity of the left ovary to the rectum, and the exposure to any irritation thence arising. This pain may be a constant dull aching, or it may be acute and occurring in paroxysms ; it is greatly aggravated by standing, and generally by walking ; indeed, in the severer cases, I have known the patient quite unable to walk.

There is generally some complaint of fulness about the iliac region, but upon careful examination I have rarely been able to satisfy myself that this was more than a sensation ; I certainly never felt any thing like a distinct tumor. There is, however, always considerable tenderness, which is extreme to the slightest touch. When the irritation is great, it may be extended to the bladder, giving rise to a desire to evacuate its contents frequently, and causing great pain in doing so. Hysterical paroxysms are by no means frequent. In two of the most violent cases of hysteria that I have seen for some time, there was extreme tenderness of the region of the left ovary, and pressure there aggravated the hysterical paroxysm.

If we make a vaginal or rectal examination, we shall most frequently discover nothing unusual, neither heat, nor tenderness, nor swelling; in a few cases, however, I have found that moving the uterus laterally caused uneasiness in the side affected. When speaking of a rectal examination in subacute ovaritis, Dr. Tilt remarks, that the ovaries are more or less painful on pressure, than they are from twice to four times their original size.* This I have not found in the affection under consideration, and it constitutes one reason for my doubting that it is the same disease as that described by Dr. Tilt.

These are principal local and direct symptoms I have observed; they vary much in degree, and are in some cases so intense as to resemble an attack of acute ovaritis. They differ also more or less according to the circumstances in which the attack occurs; and in order to elucidate this point, I shall briefly enumerate the circumstances.

1. In patients who suffer occasionally from amenorrhœa, it is not uncommon to find ovarian irritation at these periods, and not altogether confined to them. Whether the ovarian irritation be the cause of the suppression of the catamenia, or merely a symptom, is a question not easily decided. In many cases I think it is probably the primary affection, but in some others it appears to be the result of the amenorrhœa. The suffering is often considerable, and may be prolonged until the next catamenial evacuation; and if that be full and free, the pain and tenderness generally disappear.

2. Upon the sudden suppression of menstruation, it is not unusal for the ovaries to be almost instantly affected, either by the form of disease I have described, or by an acute inflammatory attack, which is more rare.

3. In dysmenorrhœa there is more or less ovarian irritation. If we examine the patient minutely as to the seat of the pain during the period, we shall find that it is principally in the region of one or both ovaries aad often accompanied by tender-

*On Diseases of Menstruation, etc., p. 79.

ness on pressure. In the majority of these cases I am inclined to think that the ovaries are secondarily affected.

4. In menorrhagia, the ovaries may apparently preserve their integrity for a long time; but if the attacks be frequent, I have generally found that these organs, one or both, become affected, and that the irritation frequently continues long after the discharge has ceased.

5. I have repeatedly seen this ovarian irritation accompany congestion and erosion of the cervix uteri, but it most frequently continues long after the discharge has persisted for some time, or after it is nearly or quite cured. The ovarian irritation, however, in these cases, very soon subsides.

6. I have already mentioned its occurrence in hysteria, both when the latter is evidently dependent upon catamenial disturbance, and when the periodical discharge is quite correct.

7. In some few cases I have recognised ovarian irritation in cases where the uterine and ovarian monthly functions were apparently accurately performed, but the patients were of a highly nervous temperament, in delicate health, and without offspring.

These various classes include, I think, all or nearly all the examples of the disease which have come under my observation. In many cases it requires care to separate the ovarian symptoms from those caused by the concurrent disease, but in other instances this distinction is quite obvious. When uncomplicated, the disorder rarely gives rise to any general or constitutional symptoms. Many of the subjects of it are delicate and weak, and of course this attack keeps them so; but ordinarily the pulse is not quickened by it, and there is neither heat of skin nor thirst. The appetite is seldom good, but it is not worse than usual, and the bowels are irregular. I have examined the urinary secretion, and have repeatedly found it scanty, acid, and occasionally mixed with mucus.

As to the *pathology* of this affection there are several points of considerable interest. I think that we can entertain no doubt that the ovaries, one or both, are the seat of the irritation: the peculiar and fixed locality of the pain, and its frequent connexion with the ovarian function of menstruation, all confirm this view. But the next question is more difficult to decide positively, viz: is the disorder an inflammatory affection of the ovaries, either acute or subacute? The disease described by Dr. Tilt certainly presents characteristics of inflammation, which I have never observed in the present disorder. The absence of tumefaction generally, and a distinct tumor always, the negative results of an examination *per vaginum* and *per rectum*, the intermitting and paroxysmal character of the attack, the absence of all the ordinary results of inflammation (as abscess, accumulation of fluid, etc.,) even in the severer cases, and the success of a certain line of treatment, are all, to my mind, very strong arguments for the non-inflammatory nature of the disease. In most of these particulars, it differs from the subacute ovaritis of Dr. Tilt. I have certainly seen some cases in which the point seemed doubtful, and it is probable that the one form of disease may, under certain circumstances, merge in the other ; but I cannot resist the conviction, that the affection I have described is essentially neuralgic, and not inflammatory.

Again, it may be asked, is this ovarian irritation the cause of the menstrual disorder or its effect, or merely a concomitant symptom ? No one acquainted with the present state of ovarian physiology could deny that the integrity of the menstrual function must be largely influenced by the condition of the ovaries. If this ovarian irritation always preceded the catamenial period, I should be inclined to attribute to it the subsequent distress ; and in many cases it appears to me that I could so trace it as the chief cause. But, in some cases, the ovarian irritation distinctly followed the menstrual disturbance or came on towards the termination of the monthly period ; and lastly, in other cases, the irritation existed with no catamenial derangement at all. Without doubting, therefore, that ovarian irritation may disturb the menstrual functions in various ways, I cannot agree with those who think that it invariably does so, nor yet with those who are inclined to attribute all menstrual disorders to deviations from the normal condition of the ovaries.

I need not occupy time by enumerating many *causes* for its production ; all those which act upon either the uterus or ovary and disturb their functions, may be considered as causes of ovarian irritation, and among these the most frequent, probably, is cold.

I believe that, in many cases, excess in sexual intercourse has given rise to it ; and I am also inclined to think, that in a few cases I have known it originate from the entire deprivation of that stimulus. For some valuable remarks on this subject, I shall refer my readers to Dr, Tilt's excellent work,* a review of which appeared in a late number of this Journal: all that he says upon this point is, I think, equally applicable to ovaritis and ovarian irritation.

The circumstances under which the attack occurs, I mean its relation to the menstrual functions, the symptoms, and the peculiar locality of the pain, render the *diagnosis* tolerably easy in most cases. It may certainly, be mistaken for intestinal irritation; but, in general, there are no other symptoms than the pain to justify such an opinion. The bowels, even if irregular, are free from irritability.

It will, however, require a little more trouble to render it certain that there is not acute ovaritis, which the tenderness might lead us to suspect. But this tenderness is *generally much greater than that resulting from inflammation;* it is a kind of a nervous tenderness which shrinks from the weight of a finger as much as from severe pressure. Moreover, in acute ovaritis, the organ is always swollen and enlarged, and it can generally be felt distinctly to be so by an internal examination.

In phlegmonous inflammation of the uterine appendages, or pelvic abscess, as it has been termed, the hard and painful tumefaction is quite plain at the brim of the pelvis, and, therefore, it cannot easily be confounded with the present disorder.

I shall not enter at any length into details of the *treatment* of this disease, inasmuch as I have only my own experience to which I can refer. The choice of remedies will be governed, to a certain extent, by the health, strength, and state of constitution of our patient. With strong, healthy women I have tried leeches to the ovarian region, with some benefit but not complete success, nor in all cases; from six to twelve may be applied at once, and repeated, if necessary, after an interval. Poultices after the leeching are of use; and indeed, when no leeches have been applied, I have seen much comfort and relief derived from repeated poulticing. With delicate women, and they are frequently the subjects of this disease, bleeding in any form has appeared to me rather injurious than beneficial.

I have tried the repeated application of small blisters with

---

*On Diseases of Menstruation, etc., p. 53.

better results than leeching. The irritation of the surface certainly relieves the pain in many cases, and, if continued, may finally cure it; but I must confess I have seen it fail repeatedly.

Anodyne liniments and anodyne plasters occasionally seem to afford relief, but they are often of little or no use; I tried anodyne enemata several times with partial success.

In two or three cases I used the tincture of aconite, applied liberally to the iliac region, but I confess the result disappointed the expectations I had formed.

Having failed in affording any relief in two or three obstinate cases, I determined to try the effect of opium applied to the upper part of the vaginal surface. I accordingly ordered some balls or pessaries to be made, somewhat in the mode of Dr. Simpson's medicated pessaries, each ball to contain two grains of opium, half a drachm of white wax, and a drachm and a half of lard. The whole, when mixed together, formed a ball about the size of a large marble, and I placed it at the upper end of the vagina by means of the speculum, leaving the patient in bed for the rest of the day. The success was quite beyond my expectation; the relief was very speedy, and in most instances complete. Even when the pain did return after a few days, a second application removed it. The tenderness disappeared with the pain, and no unpleasant consequences have resulted in any instance.

I have now tried this remedy in a considerable number of cases, and with almost invariable success. I have rarely found it necessary to bleed or blister since I first adopted this plan; and I recommend it with considerable confidence to the Profession. I may add that I have tried these pessaries in cases of dysmenorrhœa, applying one the day before the catamenia was expected, with decided benefit.

It is hardly necessary to say that, in this disease, the bowels should be regulated, and gently freed by medicine when necessary. If the appetite be bad, vegetable bitters may be given, and I have generally found it useful to combine some alkali with them.—*Dublin Quarterly Journal, Aug.* 1851.

